# Neuromuscular Anatomy and Motor Patterns at the Base of Calling Behaviour in the Female Spongy Moth *Lymantria dispar*

**DOI:** 10.3390/insects15030169

**Published:** 2024-03-01

**Authors:** Paolo Solari, Giorgia Sollai, Roberto Crnjar

**Affiliations:** Department of Biomedical Sciences, University of Cagliari, 09042 Monserrato, Italy; gsollai@unica.it (G.S.); crnjar@unica.it (R.C.)

**Keywords:** electrophysiology, morphology, terminal abdominal ganglion, muscle contraction, pheromone release, nerve recording

## Abstract

**Simple Summary:**

Female moths display a rhythmic motor pattern, a “calling behaviour”, to release sex pheromones and attract of conspecific males for mating. Pheromone release occurs through a squeezing mechanism consisting of turtleneck-like folding and unfolding of the ovipositor cuticle during its rhythmic extensions and retractions. They are under the control of the terminal abdominal ganglion (TAG). The physiology of the production and release of sex pheromones in moths has been an object of great interest. In the present study we investigate the anatomical and physiological basis of calling by using the female spongy moth *Lymantria dispar* as a model insect. Our results show that the three terminal abdominal segments S7, S8 and S9 (ovipositor) are specialized structures, containing cuticular appendages, hinges, apodemes and several large muscles, innervated by TAG nerves N4 and especially by N5. N6 mainly innervates the oviduct. We also identified a number of specific motor units from nerves N4 and N5 responsible for the ovipositor movements observed during calling. Overall, extensions and retractions of the ovipositor leading to pheromone release are sustained by a coordinated motor program, which involves the activity of a few motor units under the control of TAG nerves N4 and N5.

**Abstract:**

“Calling behaviour” is a stereotyped rhythmic motor pattern displayed by female moths, by which they emit the sex pheromone to attract of conspecific males. Calling occurs through a squeezing mechanism based on the turtleneck-like folding and unfolding of the ovipositor cuticle during its telescopic extensions and retractions. This mechanism is under the control of the terminal abdominal ganglion (TAG). By combining anatomical and electrophysiological approaches, here we studied the morpho-functional organisation of the abdominal muscles and the activity of motoneurons from TAG nerve N4-N6 as correlated to the ovipositor movements during calling in the female spongy moth *Lymantria dispar*. Our results show that the three abdominal segments S7, S8 and S9 (ovipositor) are highly specialized structures containing cuticular appendages, hinges, apodemes and several large muscles, innervated by N4 and especially by N5. N6 mainly innervates the oviductal tract. We also identified a number of motor units from N4 and N5, the spike activity of which is correlated with the ovipositor movements during calling. In conclusion, the release of sex pheromones in the female spongy moth is obtained by extensions and retractions of the ovipositor operated by a coordinated motor program, which is mainly sustained by the activity of a few motor units under the control of TAG nerves N4 and N5.

## 1. Introduction

Many rhythmic motor behaviours such as locomotion, ventilation and mastication are evoked by neural circuits, located in the vertebrate spinal cord or in the invertebrate ganglia, which are termed central pattern generators (CPGs; [1,2,3,4,5]). As a consequence of their properties and connectivity, CPGs can generate and regulate the rhythmic output also in the absence of afferent input, even though both rhythmic and non-rhythmic sensory activity are important for output compensation [5,6].

A favourable model to study CPGs in insects is “calling behaviour”, a stereotyped rhythmic motor pattern displayed by female moths, in which they extend their ovipositor to emit the sex pheromone to attract conspecific males for mating [7,8,9,10,11]. In Lepidopterans sex pheromones are produced by females in a specialized gland, which consists of epithelial cells located at the level of the soft intersegmental membrane (IM) between the 9th (ovipositor) and the 8th abdominal segments [12,13,14,15]. Once produced, the pheromone is transported through the cuticle via a special porous cuticular system to be disseminated on the IM surface. As previously defined in the sphinx moth *Manduca sexta*, calling typically consists of cycling extensions and retractions, sustained by a highly coordinated muscular activity, of the 8th and the 9th abdominal segments (at rest they are telescoped within the 7th segment) by refolding of the soft interposed IM bearing the pheromone glands [16]. Sex pheromones are detected by olfactory sensilla located mainly in the antennae, maxillary palps and ovipositor [17,18,19,20,21,22]. The olfactory sensory neurons hosted by the olfactory sensilla transduce the information carried by the pheromone molecules into action potentials which, traveling along the olfactory nerve line, reach the antennal lobes, the first centre of the brain where the olfactory information is processed [23,24,25,26,27]. The neural networks primarily involved in the control of calling behaviour and in the production and/or release of sex pheromones in moths is represented by the caudalmost terminal abdominal ganglion (TAG; [7,16,28,29,30,31,32,33,34,35]. For instance, in the Arctiid female *Utetheisa ornatrix* a neural control exerted by the TAG appears to exist in the rhythmic exposure of the sex pheromone glands during calling [28]. Other studies, carried out on the Noctuid moths *Helicoverpa zea* and *Heliothis virescens* and on the sphinx moth *Manduca sexta*, showed that pheromone glands receive neural connections from the TAG, and that electrical stimulation of the terminal nerves elicits an increase in both pheromone biosynthesis and release [31,32].

Previous studies on the spongy moth (formerly gypsy moth) *Lymantria dispar* have shown that the alternate cyclic movements of extension and retraction of the ovipositor observed during calling are controlled by the TAG, in particular by way of its three most caudally located nerve pairs IV, V and VI [29]. Calling is also disrupted or strongly depressed by transection of the ventral nerve cord (VNC) anterior to the TAG or removal of the ganglion [33,36,37]. In this moth it has been proposed that, once biosynthesized by the gland tissue located both in the dorsal and ventral epithelium of the IM, the sex pheromone is then released by the squeezing action promoted by the turtleneck-like folding and unfolding of the ovipositor cuticle during the telescopic extensions and retractions of the ovipositor [7]. In this way the sex pheromone can then reach and spread onto the cuticular surface outside the IM in discrete amounts during ovipositor retraction, to be then exposed to free air during the following ovipositor extension. More recently, it was found that the biogenic amine octopamine strongly enhances the activity of TAG nerve pairs IV-VI, either by increasing the firing rate of a number of spontaneously firing units or by recruiting new ones, thus affecting the muscle activity leading to cycling extensions of IM during calling [35].

Based upon these observations and taking into account that the CPG sustaining the calling behaviour resides within the TAG, the present study aims at elucidating the motor program which operates the cycling ovipositor extensions and retractions and thus the squeezing mechanism at the base of the pheromone release in the female spongy moth *L. dispar*. To this end, thus, the study was planned into the following phases: (1) the gross anatomy of the three caudalmost abdominal segments in the female spongy moth was first investigated in order to build a map of their neuromuscular arrangement, by highlighting the different muscles, their insertions and especially the innervation they receive from the nerves emerging from the TAG; (2) simultaneous recordings from nerves IV to VI and muscles were then performed to physiologically identify the motor units possibly responsible for the cycling ovipositor extensions and retractions observed during calling; and (3) the firing patterns of the different motoneurons projecting through each of these nerves were related to the whole ovipositor movements, in order to establish the involvement and the reciprocal contribution to calling by the different previously determined motor units.

On the whole, the present study provides new information on the motor program underlying the calling behaviour in the female spongy moth, by showing that, despite a relatively simple neuromuscular arrangement, the complexity of the movements exhibited during calling requires high coordination from a number of abdominal muscles and motoneurons projecting from the TAG via nerve pairs IV and V.

## 2. Materials and Methods

### 2.1. Insects

All experiments were performed on 2- or 3-day-old virgin female *Lymantria dispar* spongy moths (day 1 being the day of emergence), collected as pupae in a locality from central Sardinia (Abbasanta, Italy; 40°12′ N 8°47′ E). They were kept in an environmental growth incubator (24–25 °C, 70% R.H., 16 h light/8 h dark photoperiodic regime) and checked daily until adult emergence. Males and females were sexed and separated immediately after emergence to avoid reciprocal exposure. Calling females were tested between 7 and 10 h after the onset of photophase in order to reduce discrepancies due to diel periodicity in calling behaviour and pheromone release [38,39,40].

### 2.2. Anatomy

**Figure 1:**
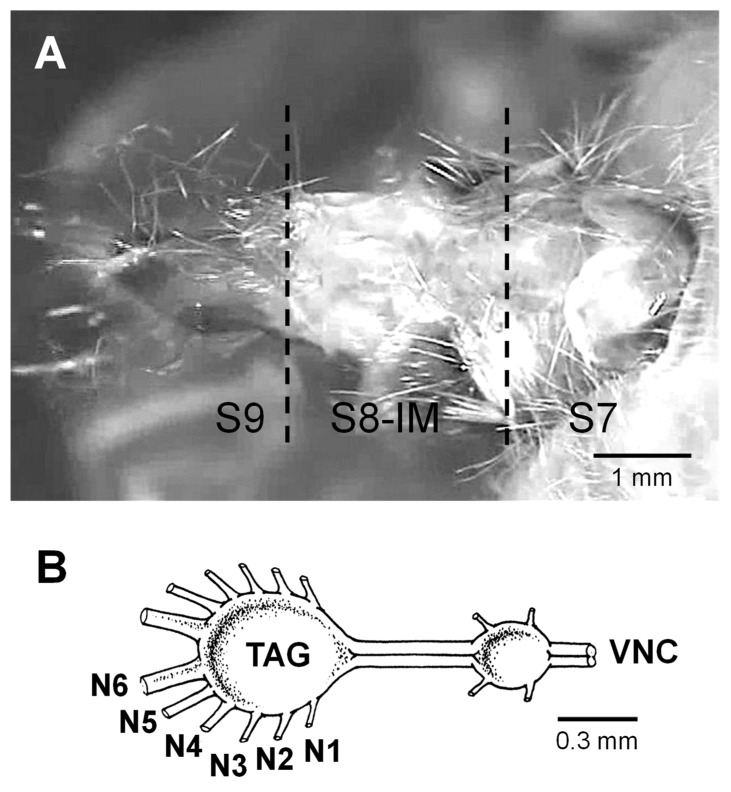
(**A**) Micrograph of the ventro-lateral view of the extended three caudalmost abdominal segments of a female spongy moth *L. dispar*, S7 (the 7th segment), S8-IM (the 8th segment including the intersegmental membrane) and S9 (the 9th segment, the ovipositor) (Modified from Solari et al. [7]). (**B**) Schematic drawing of the distal portion of the central nervous system (posterior left) of an adult female spongy moth, showing the ventral nerve cord (VNC) and the terminal abdominal ganglion (TAG) with its six peripheral nerve pairs (N1 to N6).

Light microscope analyses were performed on ice-anaesthetized specimens dissected along either the ventral, dorsal or lateral (both sides) midline of the three caudalmost abdominal segments in order to evaluate the neuro-muscular arrangement responsible for calling behaviour from all perspectives. The abdominal segments—the 7th, the 8th including the intersegmental membrane (IM) between the 8th and the 9th, and the 9th (ovipositor)—and their relative innervation from the six nerve pairs emerging from TAG will be hereafter referred to as segments S7, S8-IM and S9 (Figure 1A) and nerves N1 to N6 (Figure 1B), respectively. Drawings were made by using a “camera lucida” attachment on a stereoscope (Model Stemi 2000-C, Zeiss, Oberkochen, Germany), fitted with an optical fibre light source; twenty-six female moths were used for anatomical observations. Additional preparations, consisting of intact moths, were fixed overnight at 4 °C in 4% (*w*/*v*) paraformaldehyde in sodium phosphate buffer (0.1 M, pH 7.4), subsequently dehydrated in 10 min steps with graded ethanol of ascending concentrations, and cleared in methyl salicylate with the ovipositor pinned at various degrees of extensions and retractions. They were used to examine the macroscopic morphology and to determine the relative position of chitinous apodemes for muscle insertions.

Muscle innervation was studied in methylene blue “in vivo” (0.3% in distilled water) stained preparations, implemented by electrical stimulation of TAG nerve branches to produce contraction of the appropriate muscle. Electrical stimulations were performed according to Christensen et al. [32], with a pair of fine silver-wire bipolar electrodes; trains of two to twenty-five electric pulses were delivered by means of an electronic stimulator at a frequency of 20 Hz and the voltage amplitude was adjusted until slight contractions of abdominal muscles could be detected, typically 1–5 V. All dissections were performed in physiological saline containing (g/L): 0.7 NaCl, 0.48 KCl, 1.14 MgCl_2_, 0.11 CaCl_2_, 63.8 glucose, 0.54 KOH; final pH 6.59 [35]. The different muscles were named according to an arbitrary nomenclature.

### 2.3. Physiology

Ice-anaesthetised female moths were restrained with insect pins ventral side up on a Sylgard-coated Petri dish in saline, and a patch of cuticle was then cut from the ventral surface of the abdominal segment S7 in order to expose the TAG and the roots of its six nerve pairs. The TAG was then freed of the surrounding tissue and eggs, while its six nerve pairs were kept intact and exposed so they could be easily reached by the electrodes. Care was taken to ensure that the central nervous system, all peripheral nerves and muscles remained intact; preparations that failed to resume calling behaviour after dissection were discarded.

Extracellular recordings of spike activity from nerves N4 to N6 emerging from the TAG were made “en passant”, close to their emergency from the TAG, by way of borosilicate glass suction electrodes. These nerve pairs were chosen on the basis of the previous electrophysiological findings by Crnjar et al. [29] and Solari et. al. [35], according to which they carry most of the spike activity related to the ovipositor movements associated with calling behaviour. Recordings were preamplified and band-pass filtered (100–1000 Hz) by using a four-channel differential AC amplifier (Model 1700, A-M System, Everett, WA, USA), digitised by means of an Axon Digidata 1440A A/D converter (sampling rate, 10 KHz per channel) and stored on PC for further analyses. In each experiment the spontaneous spike activity was recorded for the following 30–40 min, only when stable for at least 2–3 min.

When access to S7, S8-IM and S9 musculature was also required for electromyographic recordings (EMGs), the mid-ventral incision of the insect cuticle was extended distally to the abdomen tip, and the cuticle pinned with the ovipositor in a fully extruded position in order to expose the different muscles. EMGs were simultaneously recorded with the spike activity of nerves N4 to N6 by way of small suction electrodes from the surface of muscle bundles (band-pass filter, 100–1000 Hz).

In a different batch of experiments, the spike activity from the same nerves was recorded and related to the ovipositor movements, that were simultaneously video-recorded by way of a Moticam 2300 (3.0 MPixel, USB 2.0) colour digital camera coupled to a stereomicroscope (Zeiss, Stemi 2000-C). Video information was provided with marks for correlation with the nerve activity during the subsequent frame-by-frame analysis (Motic Images Plus 2.0 ML software, Motic, Hong Kong, China). Resolution was set at 800 × 600 pixels and 25 frames per second were captured. The magnitude of the ovipositor movements during experiments was calculated and plotted in accordance with the spike frequency intervals considered.

Since data did not conform to normal distribution (Kolmogorov–Smirnov test for goodness of fit), the non-parametric Spearman rank test was used to establish any possible correlation between spike firing frequencies from nerves N4 to N6 and ovipositor movements, according to [41]. Analyses were carried out by using the Prism program (GraphPad Software, San Diego, CA, USA) with a 95% of confidence level (*p* ≤ 0.05).

### 2.4. Action Potential Sorting

The spontaneous spike activity recorded from each of the TAG nerves N4 to N6 was analysed by means of the Clampfit 10.0 software (Axon Instruments) [42,43,44]. Spikes in the discharges were sorted out by a threshold-based search and then their peak-to-peak amplitude (PPA) and the max decay slope (MDS) of each spike were measured as an index of their amplitude and shape, respectively, and plotted versus the time of occurrence. PPA and MDS distribution histograms were also created and a combination of both types of plots was always considered to determine the threshold for spike sorting. Therefore, spikes could be clustered and assigned to the different classes also when differing only in PPA or MDS. The relative PPA and MDS ratio of the different spikes remained conserved throughout the various experiments. This allowed the assignment of a given spike to the same class (i.e., motoneuron) throughout the experiments, regardless of the preparation and the number of different spikes elicited at any time, and frequency estimation. In the different discharges, each firing neuron was labelled with a progressive number based on the decrease of the spike PPA or, if necessary, as in the case of spikes with similar amplitude, also to decrease in MDS.

## 3. Results and Discussion

The present study describes the anatomical and physiological basis of the hitherto little-explored rhythmic motor pattern underlying the calling behaviour in the female spongy moth *L. dispar*, i.e., the complex behaviour by which female moths extend their ovipositor to emit the sex pheromone to attract conspecific males for mating [7,8,10,11].

The morpho-functional data here presented provide evidence that cycling ovipositor extensions and retractions typical of calling are sustained by a number of muscles located within the three female caudalmost abdominal segments. Their activation in the proper sequence is achieved by a coordinated neural rhythm activity produced by the TAG, in particular by motoneurons projecting through nerve pairs N4 and N5.

### 3.1. Muscles Operating the Ovipositor Movements and Relative Innervation

**Figure 2:**
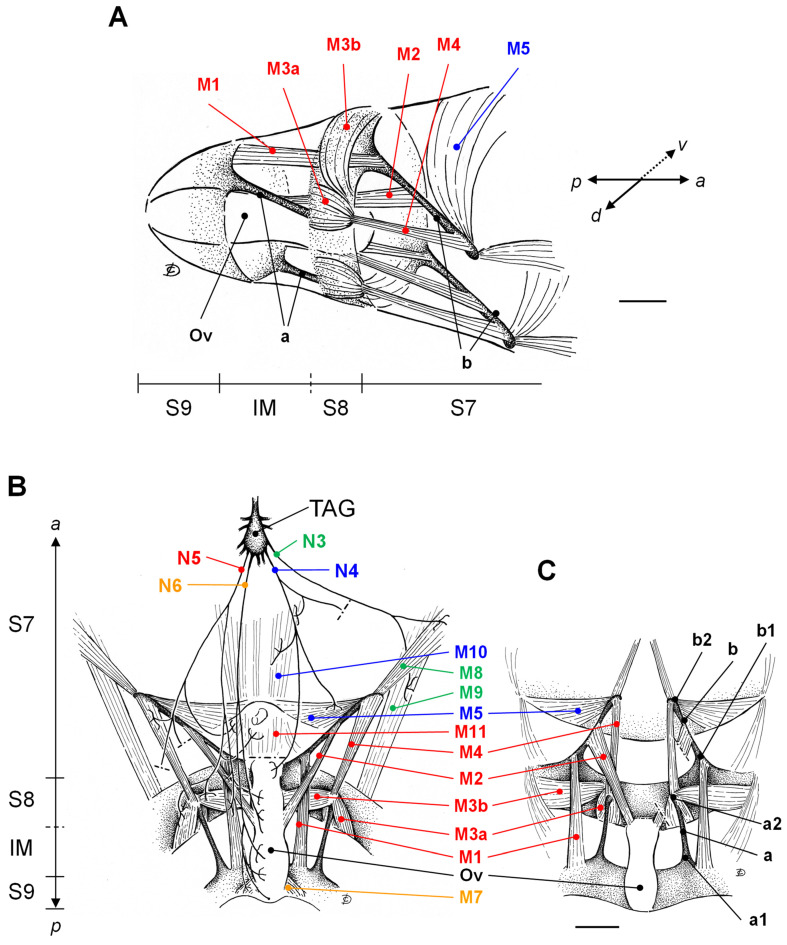
Three-dimensional (**A**) or two-dimensional ((**B**) ventral projection and (**C**) dorsal projection) schematic drawings showing the oviduct (Ov), the muscles (M1–M11), the apodemes of insertion (a, b) and respective innervations (nerves N3, N4, N5 and N6 emerging from the TAG) in the three caudalmost abdominal segments: S7 (the 7th segment), S8-IM (the 8th segment, including the intersegmental membrane IM), and S9 (the 9th segment, ovipositor). The colour code identifies the different TAG nerves and the relative innervated muscles. See the text for explanation. Bar: 1 mm (**A**) and 1.5 mm (**B**,**C**). Image orientation: a = anterior, p = posterior, d = dorsal, v = ventral.

As shown by the three-dimensional drawings of Figure 2A, in the female spongy moth the three caudalmost abdominal segments, S7, S8-IM and S9 (ovipositor), comprise cuticular appendages, hinges, apodemes and large muscles responsible for the ovipositor movements. At rest, S8-IM and S9 are telescoped within S7. Segment S9, the ovipositor, is the tip of the abdomen and externally appears as two pairs of shovel-shaped structures, the ovipositor valves. They may be swung open and closed about their hinges by contraction of muscle M7 (Figure 2B), which is innervated by a collateral branch of nerve N6. While the overall anatomical organization of the female reproductive tract is well conserved across insects [45], the ovipositor may exhibit extreme diversification levels. In fact, it is functional not only as an egg-laying guide, but its valves may also specialise into digging tools for egg deposition in the soil, such as in crickets, locusts and grasshoppers [46,47,48], or into drilling structures to reach hosts, as in the case of parasitoids [49,50,51]. Furthermore, the ovipositor can carry different types of sensilla with chemo- and/or mechanosensory function for the identification of suitable oviposition sites, as in females of *Culicoides imicola* [20], *Drosophila suzukii* [52] and *Helicoverpa assulta* [53].

In the spongy moth we found that the attachment sites for muscles operating the ovipositor movements include the body wall of segments S8-IM and, at least in part, S7, and two different pairs of robust chitinous apodemes. They offer insertion to a number of muscles, thus conveying the force to operate most of the ovipositor movements. In fact, the caudalmost apodeme pair (“a” in Figure 2C) is strongly hinged to the dorso-lateral margins of the ovipositor valves (“a1”) and dorsally extends along S8-IM, while the second, proximal pair (“b” in Figure 2C) takes origin as a lateral eversion of the rigid chitinous ring of segment S8 (“b1”) and protrudes within S7. Thus, both apodeme pairs each have a distal fixed extremity (“a1” and “b1”) and a proximal free, movable ending for muscle insertion (“a2” and “b2”). These apodeme pairs are likely to be involved in the ovipositor movements. Similarly, in the female tobacco hornworm, *Manduca sexta*, two corresponding sets of apodemes were reported to offer insertion to the muscles operating ovipositor extensions and retractions [54,55]. Instead, less homology is present with the apodeme system described in grasshoppers and locusts [47,56,57,58], which shows a higher level of complexity also in consideration of its specialization in soil digging activity for underground oviposition.

Apart from the flattened muscles covering the cuticular body wall of the abdominal segments (mainly S7), a number of muscle bundles can be easily distinguished in the female spongy moth (Figure 2B,C). Among them, muscle M1 inserts on the margin of the ovipositor valves, runs in a lateral position parallel to apodeme “a” and attaches to the fixed ending “b1” of apodeme “b”. Muscles M3a, M3b and M4 all take insertion on apodeme ending “a2”. M3a and M3b diverge from each other, the former lying on a dorsal position and the latter running towards the ventral abdominal midline. They both insert at the margin where the chitinous ring of segment S8 joins the soft cuticle of IM. Instead, muscle M4 runs in the opposite direction and finds attachment site in “b2”, thus connecting both free endings of the two apodeme pairs. Finally, the longitudinal muscle M2 emerges from the oviduct and connects it with the medial portion of apodeme “b”. Muscles M1, M3a, M3b, M4 and M2 are all innervated by nerve N5 which can be considered the main nerve sustaining the neural control of the ovipositor movements. Additionally, intrasegmental muscle M11, innervated by N5, takes origin from the margin of segment S8 (Figure 2B) and lies beneath the ventral cuticle of segment S7, in proximity of the anal opening of the insect. Two additional muscles, M5 and M10, are innervated by N4. M5 extends from apodeme ending “b1” to the body wall of segment S7, while M10 is an intrasegmental muscle which looks like an elongation of M11 and extends along the ventral midline of segment S7 up to the margin with S6.

All nerves N4 to N6 also send a dense network of collateral branches to the common oviduct, thus innervating, according to their topographical distribution, its proximal, medial and distal parts, respectively. As a whole, this topographical neuromuscular asset reflects the previous findings by which the caudally located nerve pairs N4 to N6 of the TAG carry most of the current operating rhythmic movements of ovipositor extension and retraction observed during calling [29,35]. Finally, two more muscles, M8 and M9, which are innervated by nerve N3 (Figure 2B), distally cling to apodeme ending “b2” and to the chitin of segment S8, respectively, and take proximal insertion on the body wall of segment S7.

### 3.2. Identification of Motor Units Operating the Ovipositor Movements

**Figure 3:**
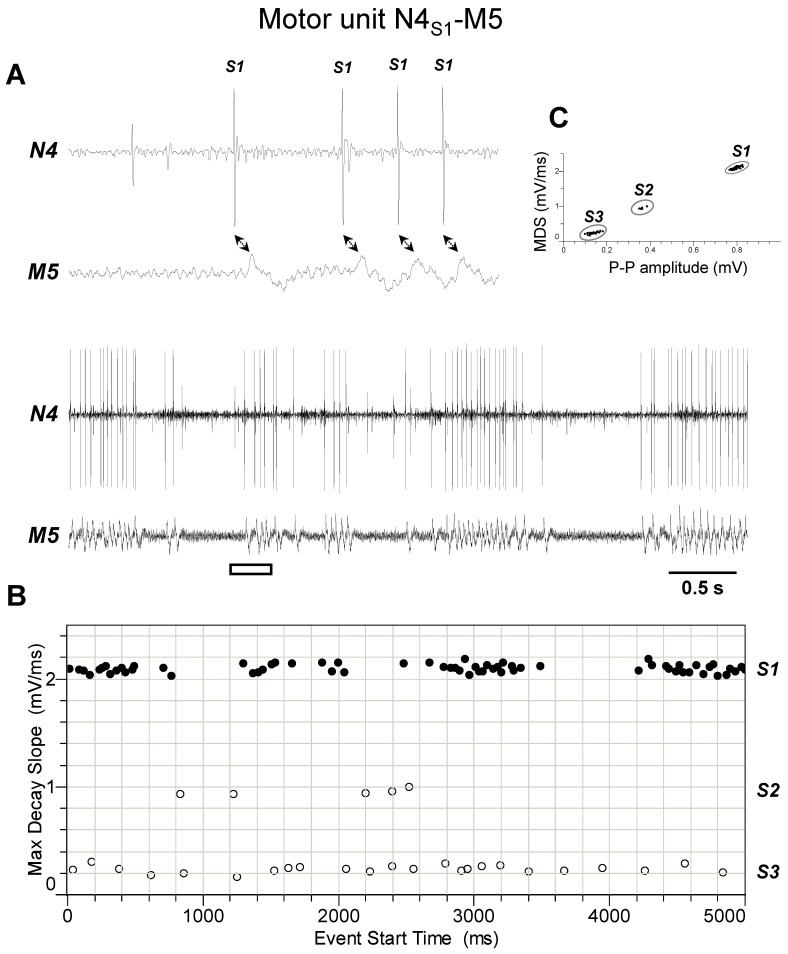
Identification of motor unit N4_S1_-M5. (**A**) Double simultaneous recording from the nerve N4 emerging from the TAG and the muscle M5 in a female spongy moth. The white bar indicates the portion of the discharge enlarged in the upper trace. Double arrows denote the presence of a phase-locked activity between spike classified as S1 from nerve N4 and twitches of muscle M5. (**B**) Distribution of spikes from the recording shown in A based on their maximum decay slope over the time of occurrence. They were assigned to the three different spike classes, S1, S2 and S3. The black dots and the hollow circles represent the nerve spikes in phase and not in phase with the muscle twitches, respectively. (**C**) Classification of spikes based on their max decay slope (MDS) as a function of the peak-to-peak amplitude. Data are representative of five experiments.

Previous electrophysiological studies on the spongy moth TAG indicated that the most caudally located nerve pairs N4, N5 and N6 carry the majority of the spike activity related to the ovipositor movements associated with calling [29]. Therefore, simultaneous recordings from these nerves and muscles located in the three caudalmost abdominal segments, S7, S8-IM and S9, were performed in order to determine which of the detected muscles is activated by each spike type from each nerve. This first allowed the physiological identification of a number of motor units possibly responsible for the ovipositor movements. Then, the recordings from the same TAG nerves were related to the video-recorded ovipositor movements in calling females, in order to understand the specific contribution of the different motor units to calling. Due to the standardised recording conditions, by plotting the spike peak-to-peak amplitude (PPA) vs. the respective max decay slope (MDS), each nerve showed a characteristic spike firing profile with fixed clusterisation. Specifically, three different clusters of spikes, attributable to the activity of three motoneurons, could be easily identified in extracellular recordings (*n* = 5) from nerve N4, as summarized by the representative experiment shown in Figure 3. Spike classes constantly differed in amplitude and shape, but the largest action potential, designated as spike S1, was constantly and exclusively phase-locked with twitches of muscle M5 in a 1:1 ratio. In other words, each spike S1 triggered a twitch of muscle M5, thus allowing unequivocal identification of the motor unit N4_S1_-M5 (i.e., nerve N4-spike S1-muscle M5). Instead, the two other smaller action potentials, indicated in the same figure as spikes S2 and S3, never appeared synchronised with the activity of any of the muscles detected within the abdominal segments considered. They are probably attributable to motoneurons targeting alternative muscles, such as those of the common oviduct, which we found receive innervation by nerve N4. In fact, it is known that in many insects the organs of the reproductive tract, in particular the oviduct, are innervated by caudalmost efferences emerging from the terminal abdominal ganglia displaying octopaminergic activity [35,59,60,61].

**Figure 4:**
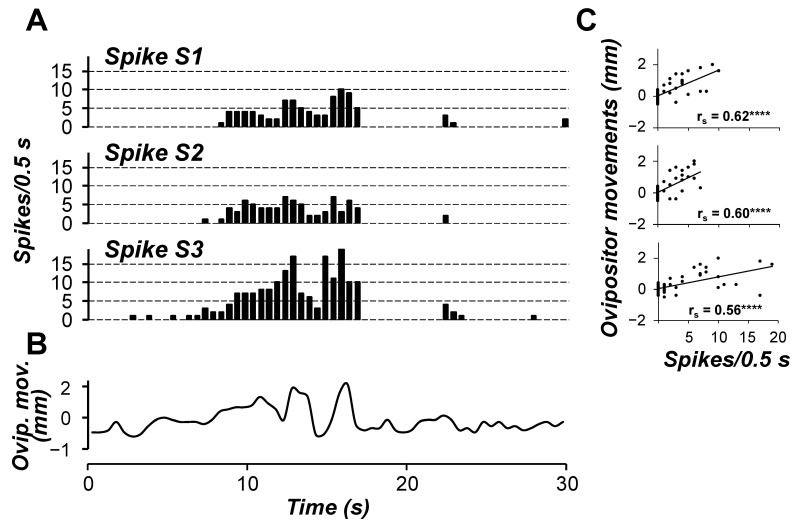
Representative time-courses of the three different spikes S1, S2 and S3 recorded from TAG nerve N4 (**A**) and of the ovipositor movements of female calling behaviour (**B**) during a 30 s interval. (**C**) Correlation between firing activity of nerve N4 and ovipositor movements. Spike frequency and ovipositor movements were calculated using 0.5 s wide bins. **** indicates a positive significant correlation (*p* < 0.0001, Spearman rank test) between spike activity and ovipositor movements (*n* = 60).

As for calling behaviour, females exhibited a considerable variation in the patterns of ovipositor movements among individuals, ranging from regular oscillatory movement without a rest even for hours to intermittent oscillations. The time course of the three different spikes recorded from N4 is shown in Figure 4. Within the time interval considered (30 s), they fired at different frequency ratios, with a minimum rate for spike S2, comprising between 0 and 7 spikes/0.5 s, and a peak of 19 spikes/0.5 s in the case of spike S3. Regardless of the spike frequency, all three cells appeared coordinated with one another, both during their bursting and silent periods, and were found to be phase-locked with the ovipositor movements. In fact, major firing activity was coupled with the largest ovipositor extension, while absent or sporadic activity was detected at small extensions or during retractions. The Spearman rank test confirmed the positive correlation between spike activity and ovipositor movements (Spearman rs = 0.62, 0.60 and 0.56 for spike S1, S2 and S3, respectively; *p* < 0.0001 in all cases). Therefore, since the spike S1 was previously shown to innervate muscle M5 (motor unit N4_S1_-M5), we infer that the ovipositor extension is driven by contraction of muscle M5 acting on the proximal ending “b2” of the apodeme “b” (Figure 2), with the aim of forcing the extrusion of segment S8-IM. In this respect, the extrusion of the terminal abdominal segments is a generally accepted prerequisite for pheromone release in moths, to expose the pheromone-producing glands, located in the soft intersegmental membrane IM of segment S8, to airstream for pheromone dispersal [13,16,62,63,64]. Conversely, no muscles possibly related to calling were found to be innervated by motoneurons firing spikes S2 and S3.

**Figure 5:**
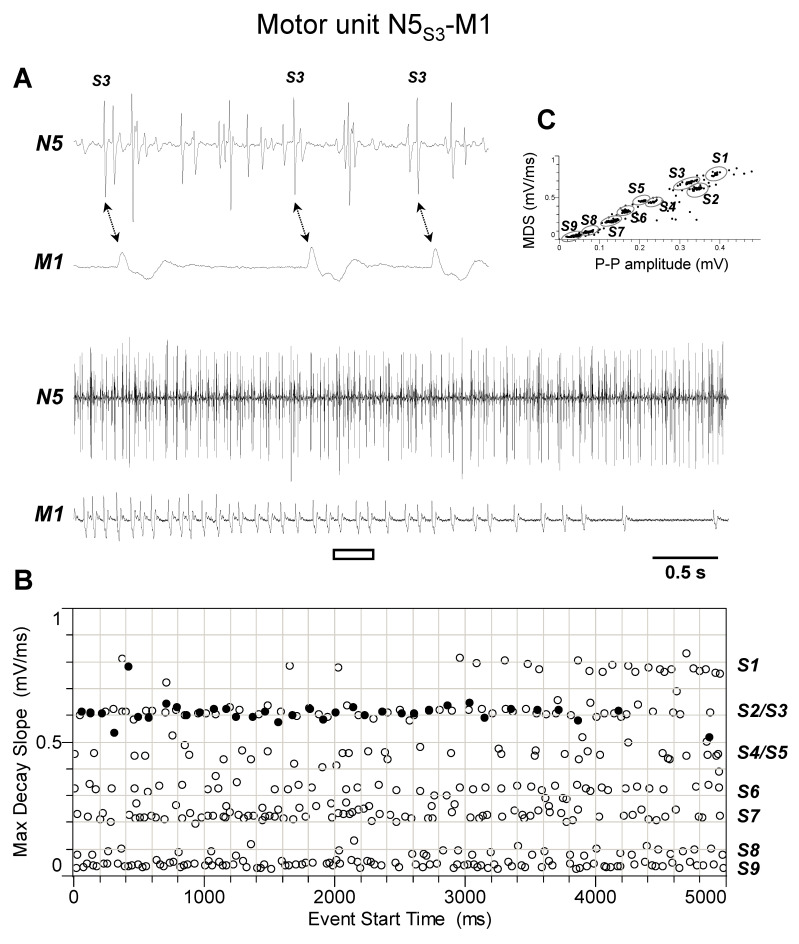
Identification of motor unit N5_S3_-M1. (**A**) Double simultaneous recording from the nerve N5 emerging from the TAG and the muscle M1 in a female spongy moth. The white bar indicates the portion of the discharge enlarged in the upper trace. Double arrows denote the presence of a phase-locked activity between spike classified as S3 from nerve N5 and twitches of muscle M1. (**B**) Distribution of spikes from the recording shown in A based on their maximum decay slope over the time of occurrence. They were assigned to the nine different spike classes S1–S9. The black dots and the hollow circles represent the nerve spikes in phase and not in phase with the muscle twitches, respectively. (**C**) Classification of spikes based on their max decay slope (MDS) as a function of the peak-to-peak amplitude. Data are representative of four experiments.

**Figure 6:**
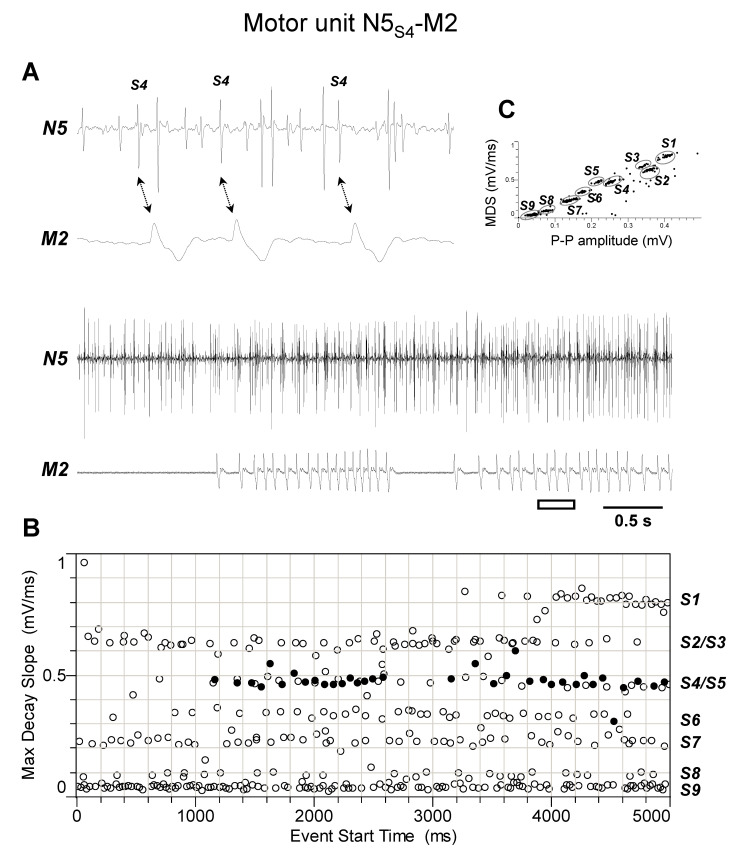
Identification of motor unit N5_S4_-M2. (**A**) Double simultaneous recording from the nerve N5 emerging from the TAG and the muscle M2 in a female spongy moth. The white bar indicates the portion of the discharge enlarged in the upper trace. Double arrows denote the presence of a phase-locked activity between spike classified as S4 from nerve N5 and twitches of muscle M2. (**B**) Distribution of spikes from the recording shown in A based on their maximum decay slope over the time of occurrence. They were assigned to the nine different spike classes S1–S9. The black dots and the hollow circles represent the nerve spikes in phase and not in phase with the muscle twitches, respectively. (**C**) Classification of spikes based on their max decay slope (MDS) as a function of the peak-to-peak amplitude. Data are representative of four experiments.

**Figure 7:**
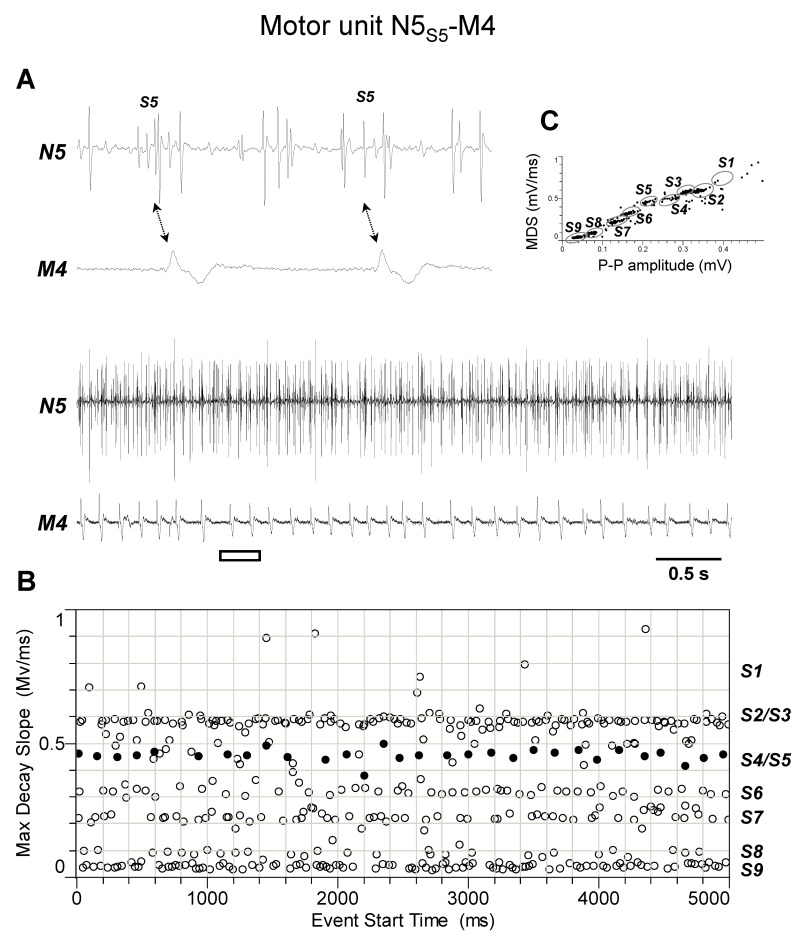
Identification of motor unit N5_S5_-M4. (**A**) Double simultaneous recording from the nerve N5 emerging from the TAG and the muscle M4 in a female spongy moth. The white bar indicates the portion of the discharge enlarged in the upper trace. Double arrows denote the presence of a phase-locked activity between spike classified as S5 from nerve N5 and twitches of muscle M4. (**B**) Distribution of spikes from the recording shown in A based on their maximum decay slope over the time of occurrence. They were assigned to the nine different spike classes S1–S9. The black dots and the hollow circles represent the nerve spikes in phase and not in phase with the muscle twitches, respectively. (**C**) Classification of spikes based on their max decay slope (MDS) as a function of the peak-to-peak amplitude. Data are representative of four experiments.

**Figure 8:**
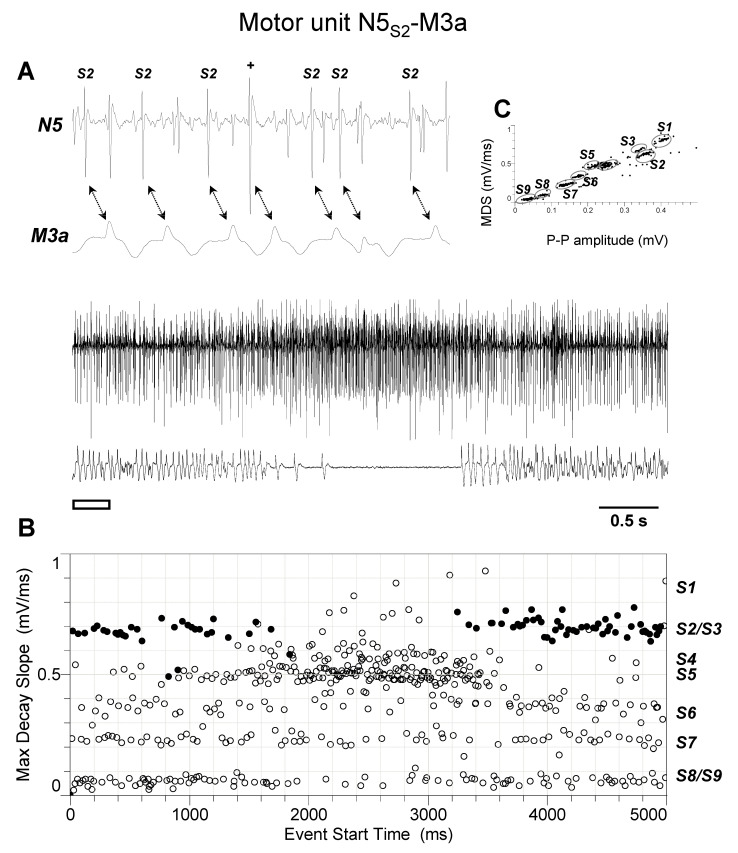
Identification of motor unit N5_S2_-M3a. (**A**) Double simultaneous recording from the nerve N5 emerging from the TAG and the muscle M3a in a female spongy moth. The white bar indicates the portion of the discharge enlarged in the upper trace. Double arrows denote the presence of a phase-locked activity between spike classified as S2 from nerve N5 and twitches of muscle M3a. (**B**) Distribution of spikes from the recording shown in A based on their maximum decay slope over the time of occurrence. They were assigned to the nine different spike classes S1–S9. The black dots and the hollow circles represent the nerve spikes in phase and not in phase with the muscle twitches, respectively. (**C**) Classification of spikes based on their max decay slope (MDS) as a function of the peak-to-peak amplitude. Data are representative of four experiments.

**Figure 9:**
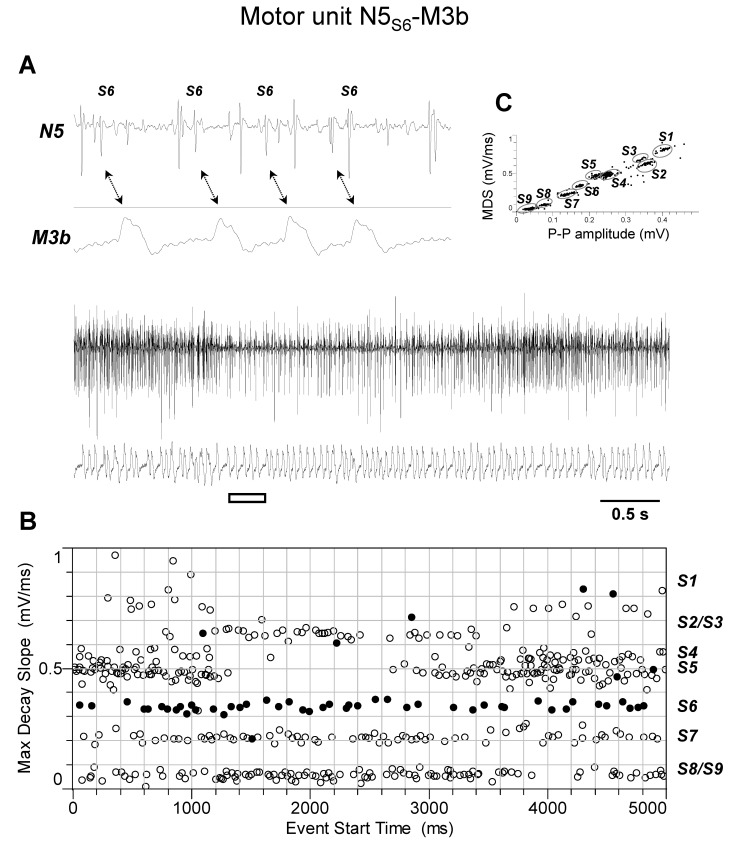
Identification of motor unit N5_S6_-M3b. (**A**) Double simultaneous recording from the nerve N5 emerging from the TAG and the muscle M3b in a female spongy moth. The white bar indicates the portion of the discharge enlarged in the upper trace. Double arrows denote the presence of a phase-locked activity between spike classified as S6 from nerve N5 and twitches of muscle M3b. (**B**) Distribution of spikes from the recording shown in A based on their maximum decay slope over the time of occurrence. They were assigned to the nine different spike classes S1–S9. The black dots and the hollow circles represent the nerve spikes in phase and not in phase with the muscle twitches, respectively. (**C**) Classification of spikes based on their max decay slope (MDS) as a function of the peak-to-peak amplitude. Data are representative of four experiments.

The discharge profiles recorded from TAG nerve N5 during the different experiments (*n* = 4) appeared more complex than those from nerve N4. Indeed, as shown by representative experiments of Figure 5, Figure 6, Figure 7, Figure 8 and Figure 9, they usually comprised the activity of up to nine different spikes, some of which resulted in time-related twitches of the abdominal muscles. In particular, spike S3 was phase-locked with twitches of muscle M1 (motor unit N5_S3_-M1, Figure 5), while spikes S4 and S5 with those of muscles M2 and M4, respectively, thus identifying motor units N5_S4_-M2 (Figure 6) and N5_S5_-M4 (Figure 7). In addition, spikes S2 and S6 were constantly phase-locked with twitches of muscles M3a and M3b, respectively, defining the other two motor units N5_S2_-M3a and N5_S6_-M3b (Figure 8 and Figure 9).

**Figure 10:**
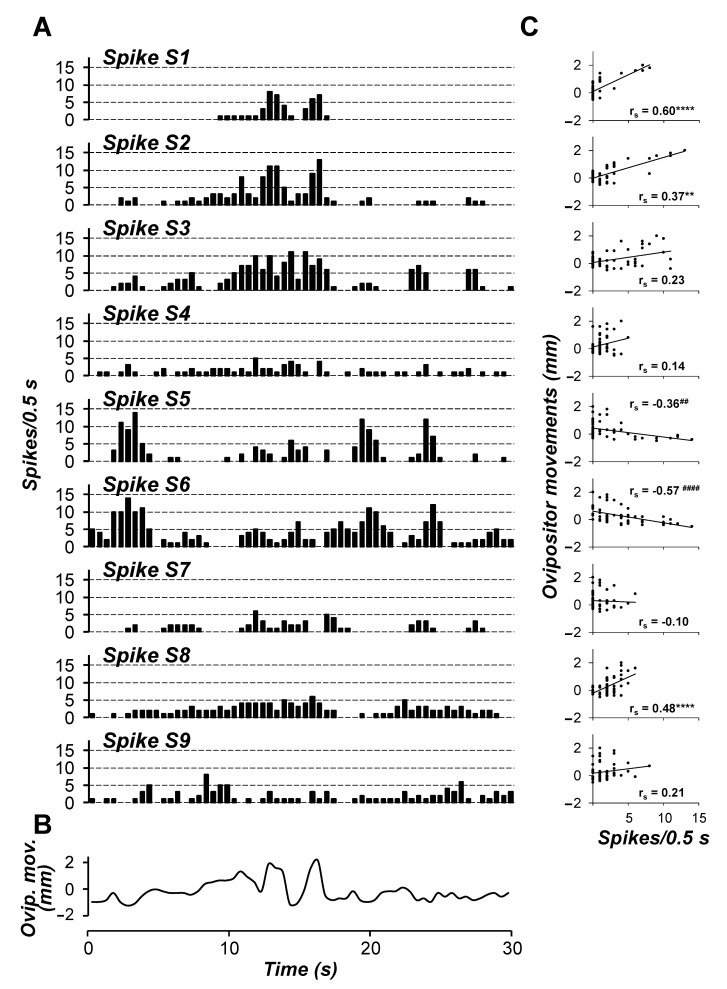
Representative time-courses of the nine different spikes S1–S9 recorded from TAG nerve N5 (**A**) and of the ovipositor movements of female calling behaviour (**B**) during a 30 s interval. (**C**) Correlation between firing activity of the different spikes S1–S9 and ovipositor movements. Spike frequency and ovipositor movements were calculated using 0.5 s wide bins. ** and **** indicate a positive significant correlation (*p* < 0.01 and *p* < 0.0001, respectively, Spearman rank test), while ^##^ and ^####^ indicate a negative significant correlation (*p* < 0.01 and *p* < 0.0001, respectively, Spearman rank test) between spike activity and ovipositor movements (*n* = 60).

When N5 recordings were related to calling (Figure 10), the spike S2 resulted positively correlated with the ovipositor extensions (Spearman rs = 0.37, *p* < 0.01). Therefore, the muscles M3a from the motor unit N5_S2_-M3a may be considered an ovipositor extensor by acting on the proximal ending “a2” of the apodeme “a” (Figure 3). In this way, M3a may force the ovipositor extrusion to distend the IM-containing glands of segment S8 and facilitate pheromone exposition to air. In this respect, the motor unit N5_S2_-M3a would act in concert with N4_S1_-M5 described above for a simultaneous extrusion of the two caudalmost abdominal segments, S8 and S9, and IM connecting them.

Unlike what was observed for nerve N4, two spikes from nerve N5, namely S5 and S6, resulted inversely correlated with the ovipositor extensions (Spearman rs = −0.36, *p* < 0.01 and rs = −0.57, *p* < 0.0001 for spike S5 and S6, respectively). This implies that the muscles M4 and M3b from the motor units N5_S5_-M4 and N5_S6_-M3b may act as ovipositor retractors and are possible antagonists of the extensor muscles M5 and M3a. In fact, once the sex attractant is dissipated during extensions of the ovipositor and S8-IM, these two segments should be retracted so that new aliquots of pheromone are squeezed onto their cuticle surface and exposed to air during the next ovipositor extension. This sequence would be repeated over and over throughout calling activity, as previously suggested for this and other moth species [7,13,63]. Two other spikes from nerve N5, S1 and S8, resulted positively correlated with ovipositor extension (Spearman rs = 0.60 for spike S1, *p* < 0.0001; rs = 0.48 for S8, *p* < 0.001) (Figure 10). Spike S1 displayed volleys of bursts in close synchronisation with extensions and spike S8 evoked a rather phasic-tonic activity along the overall time interval considered, but no abdominal muscles were identified for them. Finally, spikes S3 and S4 (and the related motor units N5_S3_-M1 and N5_S4_-M2) and spikes S7 and S9 were not correlated with any of the ovipositor movements (0.23 > Spearman rs ≥ −0.09, *p* > 0.05 in all cases).

It is to be recalled that during calling, the caudalmost abdominal segments accomplish, besides the linear, simple extensions and retractions, a complex pattern of combined movements and/or a sequence of minor, but highly coordinated rhythmical movements. This might depend upon the action of two or more sets of muscles moving the abdominal segments in different planes. A contribution to these movements by the muscles in the oviduct and/or other abdominal organs cannot be excluded, as also suggested by the finding that nerves N4 to N6 innervate different regions of the oviductal tract. In this respect, the two motor units N5_S3_-M1 and N5_S4_-M2, for which no correlation with the ovipositor extensions or retractions was found, could be responsible, at least in part, for these minor movements. In addition, other factors, such as the abdominal internal pressure that may concur in the elongation of the ovipositor, cannot be excluded, as previously reported in locusts [65].

According to results from both electrophysiological and anatomical trials, nerve N6 was only found to project to the muscle M7, which might be considered a valve constrictor rather than an ovipositor extensor or retractor, and to extensively branch the oviductal muscles. In addition, spike firing profiles recorded from N6 were complex and did not allow unequivocal classification and for this reason N6 was not considered in these electrophysiological analyses. In fact, N6 is known to be a large nerve trunk with a mixed nature, containing motor efferences, but also a large number of sensory afferences, possibly mechano- and/or chemoreceptors, running from the more caudal abdominal district and particularly from the ovipositor valves [7,47,54,66].

## 4. Conclusions

In conclusion, the results of this study add to the currently limited available knowledge of the CPG sustaining the calling behaviour in the female spongy moth *L. dispar*, by shedding light on the neuroanatomy of the caudalmost abdominal segments and on the motor patterns generated by the TAG neurons. The different muscles possibly involved in calling were identified and, for each of them, insertions on the chitinous apodemes and/or on the abdominal wall were determined, as well as their innervation by nerves N4 to N6 emerging from the TAG. A number of motor units have also been identified and related to the rhythmic ovipositor movements of extensions and retraction.

Definitely, in the female spongy moth, the release of the sex pheromone occurs through a squeezing action due to the turtleneck-like folding and unfolding of the cuticle of the intersegmental membrane of segment S8 during the telescopic ovipositor extensions and retractions operated by a coordinated motor program, which is mainly sustained by the activity of a few motor units under the control of TAG nerves N4 and N5.

## Data Availability

The data presented in this study are available upon request from the corresponding author.
